# Effects of an impermeable layer on pore pressure response to tsunami-like inundation

**DOI:** 10.1098/rspa.2021.0605

**Published:** 2022-01

**Authors:** Margaret Exton, Harry Yeh

**Affiliations:** School of Civil & Construction Engineering, Oregon State University, Corvallis, OR, USA

**Keywords:** tsunami, soil instability, overpressure, impermeable layer, centrifuge

## Abstract

Tsunami hazards have been observed to cause soil instability resulting in substantial damage to coastal infrastructure. Studying this problem is difficult owing to tsunamis’ transient, non-uniform and large loading characteristics. To create realistic tsunami conditions in a laboratory environment, we control the body force using a centrifuge facility. With an apparatus specifically designed to mimic tsunami inundation in a scaled-down model, we examine the effects of an embedded impermeable layer on soil instability: the impermeable layer represents a man-made pavement, a building foundation, a clay layer and alike. The results reveal that the effective vertical soil stress is substantially reduced at the underside of the impermeable layer. During the sudden runup flow, this instability is caused by a combination of temporal dislocation of soil grains and an increase in pore pressure under the impermeable layer. The instability during the drawdown phase is caused by the development of excess pore-pressure gradients, and the presence of the impermeable layer substantially enhances the pressure gradients leading to greater soil instability. The laboratory results demonstrate that the presence of an impermeable layer plays an important role in weakening the soil resistance under tsunami-like rapid runup and drawdown processes.

## 1. Introduction

Tsunamis occur infrequently; when generated, they are often a result of co-seismic seafloor displacements in subduction zones. They can make landfall in nearby coastal areas as well as travel long distances across oceans to damage coastal communities far away from the tsunami source. Tsunamis’ temporal and spatial characteristic scales are unique and different from the scales of other natural hazards such as riverine floods, hurricanes and earthquakes. Tsunami events can vary greatly in their magnitude and flow characteristics; hence, the potential damage is difficult to mitigate in design. The 2011 Heisei tsunami in Japan caused many well-engineered buildings and coastal infrastructure to fail. Damages caused by the tsunami may be explained by lateral hydrodynamic forces, buoyant uplift forces and foundation failures due to soil instability [1,2]. Driving mechanisms of soil instability by tsunami action are poorly understood at present and should be advanced to appropriately design critical coastal infrastructure and buildings.

**Figure 1.**
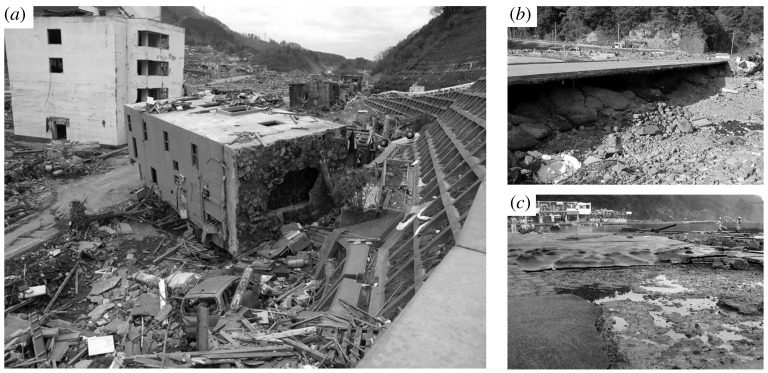
Photographs taken after the 2011 Heisei tsunami in Japan: (*a*) a four-story, reinforced concrete building supported by 32 piles was toppled and washed inland for 30 m [1] in the town of Onagawa ($38^{\circ }$ 26’ 33” N, $141^{\circ }$ 26’ 44” E) (Photo by Shunichi Koshimura); (*b*) an undermined road (1.5 m deep) along the harbour of Tohni ($39^{\circ }$ 12’ 30” N, $141^{\circ }$ 53’ 13” E); (*c*) a damaged pavement of the wharf in Yori-iso-hama ($38^{\circ }$ 23’ 22” N, $141^{\circ }$ 31’ 32” E).

There is evidence from field observations after the 2011 Heisei tsunami event that the presence of an impermeable layer in a soil bed plays a significant role in the enhancement of scour, undermining foundations, and detaching pavements during tsunami inundation [1]. The 2011 tsunami event is the first where the toppling of several reinforced concrete buildings was observed. The toppled building shown in figure 1*a* retained its structural integrity and was pushed inland from its original location, indicating that the failure must have occurred during the runup phase of tsunami inundation. Additionally, it has been reported that a relatively rapid reduction of overburden load on the soil surface during tsunami drawdown can contribute to structural damage [1,3]. The effect of tsunami-drawdown could have severely undermined paved roads such as that shown in figure 1*b*. As another example of damage induced by soil instability, figure 1*c* shows the peeled off surface of a wharf (foreground) and the large, bubble-like deformation of a pavement (background).

Studying tsunami-soil interactions is formidable because tsunami-induced overland flows are highly transient and turbulent with substantial speeds and depths. Additionally, soil is a multi-phase particulate medium that can deform permanently when subjected to small strain. Owing to the unpredictability and rarity of tsunami occurrence, soil response measurement during an actual tsunami event is impractical. It is necessary to resort to a controlled laboratory study. Traditional water-wave-tank experiments have a significant drawback due to scale effects (i.e. changes in dynamic characteristics of a phenomenon due to smaller geometric scales of the model). Despite the substantial scale effects involved, laboratory experiments to study effects of tsunami loading on soil stability have been performed previously [4,5]. Yeh *et al.* [4] conducted their experiments in a water-wave tank to study tsunami-induced scour around a vertically erected cylinder (50 cm in diameter) placed on a sloping sandy beach. They reported an occurrence of soil liquefaction around the shoreward side of the cylinder wall during the drawdown resulting in the formation of a large scour hole. They explained that, as the water level subsides rapidly, the pressure on the surface of the soil bed decreases, creating a vertical pressure gradient within the soil and decreasing the effective vertical stress (i.e. a measure of inter-soil-particle contact force) within the soil. Using their data, a prediction model for tsunami-induced soil instability was proposed by Tonkin *et al.* [6], who demonstrated that the upward pore-pressure forces could indeed cause momentary liquefaction due to the rapid reduction of overburden load on the soil surface. Tonkin *et al.* [6] claim that momentary liquefaction occurs when the pressure gradient reaches the buoyant unit weight of the soil. Abdollahi & Mason [7] developed a soil response model applicable to tsunami loading, and their numerical simulations confirm that the drawdown process induces instability at the soil surface due to the pore pressure gradient. Enhanced soil instability during the drawdown phase of a solitary wave is also confirmed by Young *et al.* [5]. Note that similar momentary liquefaction is known to occur at short time scale under the trough of a water wave when the pressure from the preceding wave crest is released from the soil [8]. On a much longer time scale, momentary liquefaction is known to occur in dams and embankments due to a rapid reservoir drawdown [9,10].

In the present study, we quantitatively explore mechanisms of soil instability associated with a tsunami-like inundation process—both its runup and drawdown—through the laboratory experiments. To mitigate the scaling issues, we perform the experiments imposing a centripetal acceleration to the scaled-down model with the use of a large centrifuge facility. By increasing the body force on the model using a centrifuge, the magnitudes of field-scale flow speed and pressure on the soil surface can be realized in the model. Additionally, as discussed in §2, the use of viscous fluid to saturate the soil enables better reproduction of the field-scale response in the pore-pressure dissipation in the soil. Although not common, centrifuge apparatuses have been used to study soil response to water waves previously. The first reported comprehensive centrifuge experiments are by Sassa & Sekiguchi [11] for their study on soils under progressive wave action. There have been a few other previous attempts to use a centrifuge for the study of tsunamis but they primarily aimed to demonstrate the engineering performance of coastal structures during the runup stage of tsunami inundation [12–15].

## 2. Background

Mechanisms of soil response to tsunami inundation are difficult to study experimentally due to the scale of the problem. To achieve the dynamic similitudes in hydrodynamics, the Reynolds $R_{e}$, Froude $F_{r}$, Euler $E_{u}$ and Rossby $R_{b}$ numbers of the scaled-down model must match those of the field condition. When this is achieved, the non-dimensionalized Navier–Stokes equation that represents the field becomes identical to the model. In a reference frame revolving at a rotation rate of Ω, the non-dimensionalized Navier–Stokes equation at the radial distance r from the rotating frame axis can be derived (for example, from [16])

2.1 $$\frac{\partial u}{\partial t}+u\cdot \nabla u+R_{b}^{-1}\Omega \times u=-E_{u}\nabla p+R_{e}^{-1}\nabla^{2}u+F_{r}^{-2}\nabla \left(\Phi_{g}+\frac{1}{2}\Omega^{2}r^{2}\right),$$

where u is the fluid velocity, t is time, p is pressure and $\Phi_{g}$ is the gravity potential: they are all normalized quantities. As discussed in §3, the primary flow direction in the model of centrifuge experiments is parallel to the axis of rotation; therefore, the third term on the left-hand side of (2.1) is unimportant—this term represents the Coriolis effect. For the field condition, the Rossby number $R_{b}$ is very large, hence this term is also negligible. The last term on the right-hand side of (2.1) represents the net body force G per unit mass. In the field, this quantity is the gravitational acceleration g, while in the centrifuge the body force is dominated by the centripetal acceleration in the radial direction, which is conveniently presented by G=Ng, in which g=|g| and G=|G|.

To study the soil response, we consider a governing equation for soil dynamics. Here we use a basic theory represented by the Terzaghi equation [17]: it is a model for the dissipation of excess pore pressure $p_{e}$ based on the assumption of a stable soil lattice structure. Non-dimensionalizing the Terzaghi equation

2.2 $$\frac{\partial p_{e}}{\partial t}=T_{z}\nabla^{2}p_{e},$$

yields the Terzaghi number $T_{z}$, which must be same for the model and the field conditions to achieve similitude. Note that the excess pore pressure is defined by $p_{e}=p+\rho Gz$, in which ρ is the fluid density and z is the coordinate from the soil surface in the direction opposite to G. Furthermore, for the condition at the soil surface, we consider matching the Shields number Θ, which represents the ratio of the fluid-induced shear force to the body force of the soil grains. Consequently, to analyse the field conditions by performing laboratory-scale experiments, the following numbers for the field and the model need to match:

2.3 $$R_{e}=\frac{\rho u_{0}L_{0}}{\mu },\;F_{r}=\frac{u_{0}}{\sqrt{GL_{0}}},\;E_{u}=\frac{p_{0}}{\rho u_{0}^{2}},\;T_{z}=\frac{\kappa t_{0}}{\mu L_{0}^{2}}\frac{E_{v}}{1-\phi },\;\Theta =\frac{\tau_{0}}{Gd_{0}\rho (S_{g}-1)},$$

where $L_{0}$, $t_{0}$, $u_{0}$, $p_{0}$ and $\tau_{0}$ are representative scales of the length, time, velocity, pressure and shear stress, respectively; $d_{0}$, $S_{g}$, $E_{v}$, κ, and ϕ are the soil grain size, specific gravity, bulk modulus of elasticity, intrinsic permeability and porosity of the soil, respectively; μ represents the fluid viscosity. For the Shields number Θ, we assume the flow is fully turbulent so that the flow-induced shear stress on the soil surface can be approximated by $\tau_{0}∼fu_{0}^{2}/8$, where f is Darcy’s friction factor [18]. In spite of the foregoing requirements to create equivalent dynamic conditions between the model and the field, it is difficult to match all of the numbers because some of the parameters (e.g. ρ, μ, G) are difficult to control. For example, the Reynolds number $R_{e}$ and the Froude number $F_{r}$ cannot be matched simultaneously unless the values of μ and G are properly controlled. This is called the scale effect in the model [18].

To examine soil response in a scaled-down model, we use soils equivalent to those in the field. This is because the use of scaled-down soil grains in the model causes problematic outcomes in soil response. According to Kutter [19], soil’s interparticle contact forces depend on stresses and the number of contacts per area, which depends on the absolute particle size. Therefore, it is important to use the same soils in both the field and the model: for example, the value of $d_{0}$ for the model and the field should be the same. It is noted, however, this constraint causes an unavoidable mismatch in the Shields number Θ, as discussed below.

For a model of 1/N the field geometric scale, stress in soils can be equivalent to that in the field when the body force is increased by N times the gravity. Under the hypergravity condition, the Euler number $E_{u}$ of the model matches that of the field; consequently, stresses in the field can be realized in the model. This is the primary advantage in using a centrifuge. Scaling considerations for 1/N scaled models in the Ng centrifuge environment are well established to study geotechnical problems [19–25]. For a more detailed discussion on scaling this problem in a centrifuge, see Exton *et al.* [20].

The present experimental study reported herein is for gravity-driven flows; hence, it is necessary to match the Froude number $F_{r}$. Imposing G=Ng in a 1/N geometric scale model, the flow speed in the model is identical to that in the field. While the Euler number $E_{u}$ is matched, the Terzaghi number $T_{z}$ would be N times greater in the model than in the field; the Shields number Θ and the Reynolds number $R_{e}$ for the model would be 1/N the field condition. To mitigate the scale effects in Terzaghi number $T_{z}$, a viscous fluid (N times larger viscosity than water) can be used to saturate the soil. This treatment is practical since a relatively small soil specimen is used in the hypergravity experiments. Saturating the soil with a viscous fluid can match the Terzaghi number, assuming that κ, $E_{v}$ and ϕ remain constant and (2.2) is valid. On the other hand, increasing the viscosity of the fluid worsens the Reynolds number mismatch in the overland flow. To circumvent this, we keep water as the surface-flow fluid. It is anticipated that the use of a different fluid for soil saturation makes little difference in shear stress on the soil surface because the bed shear stress is proportional to the velocity squared and independent of fluid viscosity for a sufficiently high Reynolds-number flow. Furthermore, it is reasonable to assume that the pore-pressure field within the soil remains unaffected as long as the fluid density remains the same.

Even with the foregoing treatments, there remains mismatch in the Reynolds number and the Shields number. None the less, the degree of mismatch in Reynolds number is substantially reduced by the use of a centrifuge: N versus $N^{3/2}$ with and without use of centrifuge, respectively. The mismatch in the Shields number Θ remains the same with or without the use of the centrifuge. In short, realistic tsunami flows and loading conditions can be achieved by use of a centrifuge due to the improved dynamic similitudes by controlling the body force combined with the use of a viscous saturating pore fluid.

To evaluate soil instability, the concept of effective vertical stress $\sigma^{\prime }$ is used in the geotechnical engineering field, which represents the net interparticle contact forces per unit area: i.e. $\sigma^{\prime }=\sigma -p$, where σ is the vertical component of the total stress and p here represents the pore pressure. (For brevity, from hereinafter, we term σ and $\sigma^{\prime }$, total stress and effective stress, respectively, without stating ‘vertical component.’) It is cautioned that the notion of effective vertical stress is not exact, because soil ‘stress’ is determined by the net force divided by a finite (not infinitesimal) area and soil’s interparticle contact occurs in a finite area but not at a ‘point.’ In other words, the validity of continuum hypothesis is in question for a soil medium. Therefore, the effective stress should be considered as an approximate indicator for soil instability.

Tsunami inundation creates variable overburden loads on the soil surface. Following Yeh & Mason [27], it is convenient to represent the effective stress in terms of the ‘excess’ pore pressure $p_{e}=p+\rho Gz$ and the ‘excess’ total stress $\sigma_{e}=\sigma +\rho_{\text{sat}}Gz$, where $\rho_{\text{sat}}$ is the bulk density of the saturated soil skeleton. Note that G=g in the field and G=Ng for the centrifuge condition. The effective stress in the soil medium can be written as

2.4 $$\sigma^{\prime }=\sigma_{e}-p_{e}-(\rho_{\text{sat}}-\rho )Gz.$$

Note that, prior to tsunami inundation, both the excess pore pressure and the excess total stress are nil everywhere in a fully saturated soil domain. Also note that vanishing effective stress $\sigma^{\prime }\to 0$ implies the state close to no net interparticle contact forces on the soil grains, i.e. an approximate indicator for the state of soil liquefaction.

The vertical gradient of excess pore pressure alters the effective body force on soil grains. The local soil grains become free from resistance when the vertical upward gradient in pore pressure reaches the buoyant specific weight of the saturated soil:

2.5 $$\frac{\partial p_{e}}{\partial z}\to -(\rho_{\text{sat}}-\rho )G.$$

Under water-flow loading on the soil surface, it was found empirically (e.g. [6,28]) that the soil becomes unstable when the effective stress becomes approximately one-half the equilibrium state: namely, $\partial p_{e}/\partial z\to -\frac{1}{2}(\rho_{\text{sat}}-\rho )G$. It is noted that, unlike (2.4), (2.5) is independent of the initial and boundary conditions. Tonkin *et al.* [6] used (2.5) to study scour formation around a vertical cylinder by tsunami-like runup and drawdown actions. Furthermore, Mason & Yeh [29] discussed the advantages of (2.5) to investigate soil response under transient loadings. Nonetheless, in the present study, we use (2.4) to evaluate the soil instability because accurate pore-pressure gradients are difficult to obtain in the small soil specimen used in the centrifuge experiments. Specifically, a dense vertical array of pore-pressure transducers is impractical to install, as described in §3.

## 3. Experimental set-up

**Figure 2.**
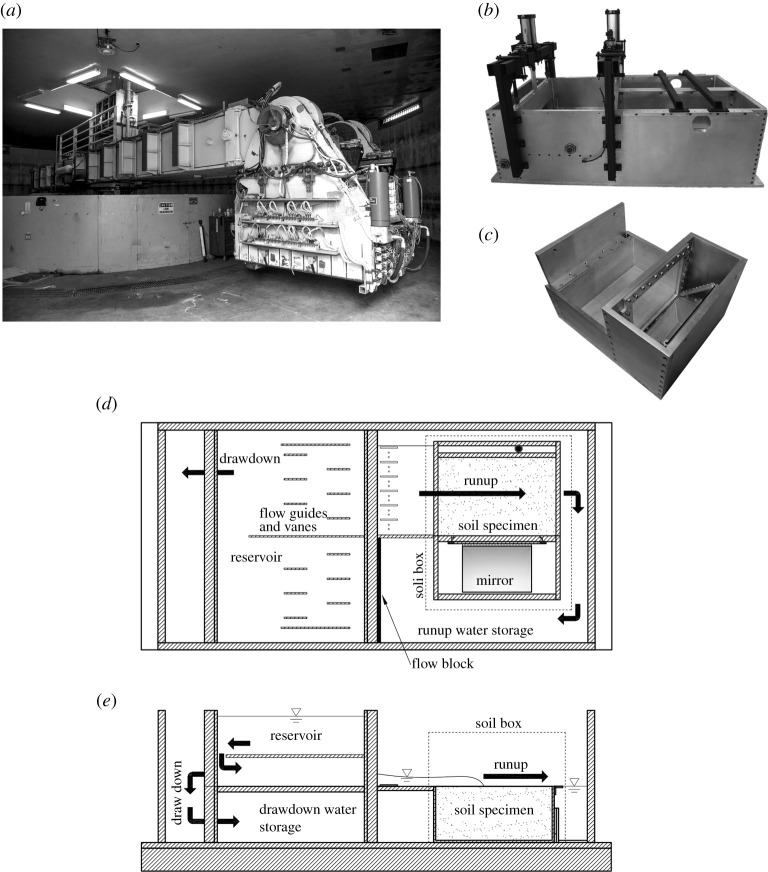
Centrifuge equipment that consists of (*a*) a 9.1 m radius geotechnical centrifuge, (*b*) a tsunami-generation container installed in the bucket of (*a*), and (*c*) a soil-specimen box installed in (*b*). Two pneumatic actuators seen in (*b*) are used to lift the discharge gates to initiate the runup phase and the subsequent drawdown phase. The angled mirror and the observation window next to the soil specimen box are seen in (*c*) on the right-hand side of the image. Schematic drawings of the container (1.93 m long, 0.94 m wide and 0.58 m deep) are depicted in (*d*) plan view and (*e*) elevation view: filled dots represent the location of water-pressure transducer to measure the flow depth. Placement of the soil-specimen box (*c*) (0.520 m long, 0.345 m wide, 0.230 m deep) is marked with the dashed line in both (*d*,*e*).

The experiments are performed in the 9.1 m radius geotechnical centrifuge at the University of California, Davis: see figure 2. When the centrifuge rotates, the bucket at the end of the arm swings outward by the centrifugal effect. The tsunami generation container (1.93 m long, 0.94 m wide and 0.58 m deep) that was specifically designed and constructed to generate realistic tsunami inundation, is installed in the centrifuge bucket. The container is equipped with two gates; one is to create the runup flow and the other to create the drawdown flow by lifting the gate with a pneumatic actuator, as seen in figure 2*b*. The soil specimen box (0.520 m long, 0.345 m wide, 0.230 m deep) is installed in the container. The soil box is designed for optical observations from an adjacent compartment that is sealed from water. This compartment houses an angled, front-surface mirror that allows an elevation view through the window from above: see figure 2*c*. Schematics of the container and the soil specimen box are shown in figure 2*d*,*e*. Because the experiments are performed under substantial body force induced by the centrifuge, the apparatus is constructed to be rigid; for example, the outside walls of the container are made of 31.75 mm thick aluminium plate and the pneumatic actuator produces 8.8 kN of force to lift the gate under enhanced body force. Detailed descriptions of the laboratory apparatus can be found in Exton [30].

**Figure 3.**
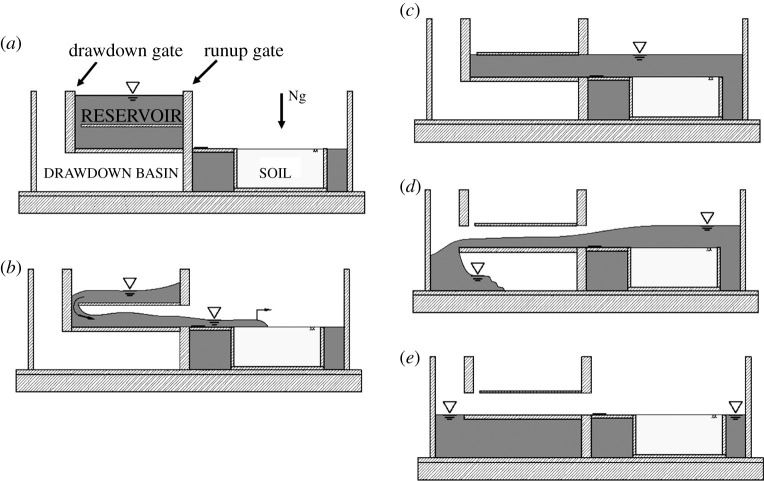
Schematics of operation of the centrifuge apparatus that applies a tsunami load to a soil specimen: (*a*) initial condition; (*b*) the runup flooding stage is established by opening the ‘runup gate’ and discharging fluid onto the soil specimen; (*c*) the fully inundated condition is maintained for 10 min to establish equilibrium; (*d*) the drawdown stage is established by opening the ‘drawdown gate’ to induce the receding flow; (*e*) final condition.

Figure 3 depicts sketches of the experimental operation from the initial to final condition. Tsunami inundation is established by opening the ‘runup gate’ and discharging fluid onto the soil specimen (figure 3*b*). With the aid of vanes (figure 2*d*), the flow is uniformly released in the portion of the container where the soil specimen is placed. The flow is then directed around and behind the observation compartment after hitting the end wall of the container. The discharged water continually flows into the space of runup water storage (figure 2*d*). The fully inundated condition (figure 3*c*) is then maintained for 10 min to establish equilibrium prior to opening the ‘drawdown gate’ to induce the receding flow (figure 3*d*). When the drawdown gate is opened, the water is rapidly drained and stored underneath the reservoir. The final condition is when all overburden is removed from the soil (figure 3*e*). During the centrifuge flight, both runup and drawdown flow directions are aligned with the axis of centrifuge rotation. Consequently, the Coriolis effect (the term involved Ω×u in (2.1)) is negligible for the flows.

We set the centripetal acceleration to 40 g (the scale factor N=40) with a model scale that is 1/40 of the field condition. Under the 40 g condition, the soil model is equivalent to a relatively large soil domain in the field scale: *viz*., 20.8 m long, 13.8 m wide and 9.2 m deep. The target tsunami condition in the field is a maximum flow depth of 5 m and a maximum runup flow speed of $10\;{\text{m s}}^{-1}$, which are considered to be realistic based on historical tsunami data: see for example the database provided by NOAA [31]. Such flow characteristics correspond to a maximum flow depth of 125 mm in the 1/40-scaled centrifuge model.

Soil used in the experiments is a fine-grained sand (Ottawa F-65): a mean grain-size diameter $d_{0}=0.21\;\text{mm}$ and a specific gravity $S_{g}=2.65$, and the model was constructed with porosity ϕ=0.38, corresponding to a relative density of 58%. Additional details of the soil properties can be found in [32]. The buoyant specific weight of the soil skeleton is $\gamma_{b}=(\rho_{\text{sat}}-\rho )Ng=403\;{\text{kN m}}^{-3}$ in which $\rho_{\text{sat}}=2026\;{\text{kN m}}^{-3}$ is the bulk density of the saturated soil skeleton. The soil specimen was fully saturated with a mixture of water and hydroxypropyl methylcellulose to establish a pore fluid with a viscosity 40 times that of water and to satisfy the Terzaghi number $T_{z}$ for the dynamic similitude of pore-pressure dissipation. While viscous fluid is used for the pore fluid in the soil, we use water to impose the flow on the soil surface to circumvent worsening the Reynolds-number mismatch, as discussed in §2. Note that this viscous-fluid density is essentially the same as water [30].

Considering that the order of magnitude of a realistic tsunami runup is a flow speed of $10\;{\text{m s}}^{-1}$ and a 2 m water depth, and the order of magnitude for the drawdown is a flow speed of $2\;{\text{m s}}^{-1}$ with a 5 m inundation depth, the corresponding scales for the N=40 model are listed in table 1. Also presented are the values of Reynolds number ($R_{e}$), Froude number ($F_{r}$), Euler number ($E_{u}$), Terzaghi number ($T_{r}$) and Shields number (Θ). As discussed in §2, all of the parameters match for dynamic similitude, except the Reynolds and Shields numbers. The Reynolds number for the model is sufficiently large so that viscous effects do not play a major role. Recall that this is an advantage of the hypergravity experiments since the Reynolds number is $N^{1/2}$ times larger than the conventional laboratory experiments. However, the Shields number mismatch remains the same as conventional experiments. The Shields number in the model is close to critical (i.e. the incipient sediment motion occurs when $\Theta ≲O(10^{-1})$), while the value for the field condition is large. Despite the mismatch of the interface condition at the soil surface, it is evident that the hypergravity experiments can create more realistic tsunami runup and drawdown loading in the model than in the conventional laboratory experiments.

**Table 1.** Flow characteristics for the field conditions for tsunami runup and drawdown, and the corresponding hypergravity model with N=40. For soil properties, we use $d_{0}=0.21\;\mathrm{mm}$, Sg=2.65, and take the soil depth at the underside of the impermeable layer. The loading times are estimated from the experiments. To estimate the Terzaghi number $T_{z}$, we used $E_{v}∼2.3\times 10^{3}\;\mathrm{kPa}$: this yields an empirical value obtained for fine sands [26]. To estimate the Shields number Θ, we assume Darcy’s friction factor f=0.01 [27].

	runup		drawdown	
	field	laboratory	field	laboratory
velocity, $u_{0}\;({\mathrm{ms}}^{-1}$)	10	10	2	2
flow depth, $L_{0}\;(m)$	2	0.05	5	0.125
soil depth, $L_{0}\;(m)$	1.2	0.03	1.2	0.03
loading time, $t_{0}\;(s)$	20	0.5	120	3.0
$R_{e}$	$2\times 10^{7}$	$5\times 10^{5}$	$1\times 10^{7}$	$2.5\times 10^{5}$
$F_{r}$	2.0	2.0	0.3	0.3
$E_{u}$	0.2	0.2	12.5	12.5
$T_{z}$	1.0	1.0	6.25	6.25
Θ	36	0.9	1.4	0.04

It is crucial to fully saturate the soil prior to the experiment because a small amount of air bubbles (or even dissolved air) could alter the soil response significantly [33]. Saturation is achieved by the following steps. First, vacuum is applied to the soil model to remove the air. Then, the model is flushed with carbon dioxide because it is 50 times more soluble in water than nitrogen. This procedure is repeated twice, prior to introducing de-aired pore fluid slowly in the soil specimen. After the specimen is completely inundated, the vacuum is slowly released. Any small amount of low-pressure gas trapped in voids compresses and dissolves in the de-aired fluid. According to Kutter [34], the preceding procedure theoretically results in full saturation.

Instruments deployed in the experiments are miniature pore-pressure transducers with the sensor head diameter of 7 mm (Keller’s 2Mie), total normal-stress transducers with the sensor head diameter of 50 mm (Tokyo Sokki Kenkyujo, model KDE-PA) and a water-pressure transducer (Schaevitz Sensors US66X-00000X-030PA). All sensors are sampled at 5000 Hz and measure the pore-pressure changes in the soil, the total stress changes and the water pressure on the surface of the soil, respectively. Note that the relatively large sensor head size of the normal-stress transducer is necessary to measure the mean stress created by the soil particles.

A high-speed high-resolution video camera (Photron AX-100) is used to record the flow using the mirror installed in the observation compartment (figure 2*c*). We set a rate of 3600 frames per second with 1024×1024 pixel resolution. Note that the high-speed video camera is needed because the model time is scaled N times faster than that of the field phenomenon.

**Figure 4.**
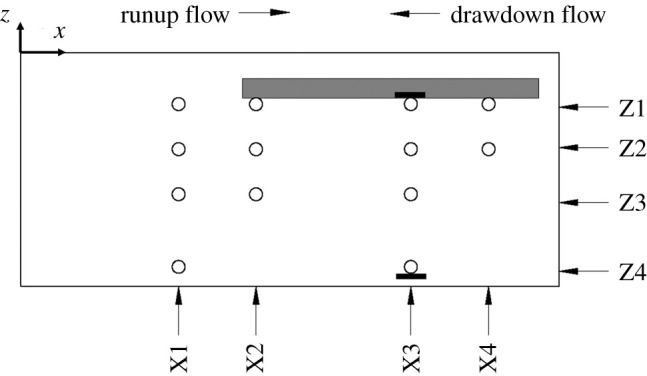
Elevation view of the soil specimen set-up within the specimen box, which is a part of the apparatus shown in figure 2*d*,*e*. ∘: locations of pore-pressure sensors that are placed along the centreline of the soil specimen box. The coordinates of pore-pressure sensors are listed in table 2. The z-axis points upward from the soil surface, and x points horizontally from the edge of the soil specimen box. Total normal-stress transducers are placed at the same location as the pore-pressure sensors at X3Z1 and X3Z4, as depicted with the thick black bar. The location of the water-pressure transducer is not shown here because it is placed on the soil surface at X3 along the sidewall of the soil box, as shown in figure 2*d*. The embedded impermeable layer is shown by the shaded area: the top surface is at z=−30 mm in the soil and the leading edge is at x=220 mm.

Two sets of experiments were performed: one with and one without placing an impermeable horizontal layer in the soil. The impermeable layer is a 29 cm by 34 cm acrylic plate with a thickness of 1.8 cm that covers the downstream half of the soil specimen. The layer spans the lateral breadth of the soil specimen and is embedded 3.0 cm below the soil surface, as shown in figure 4. The layer contains an embedded total normal-stress transducer with the sensor head flush to its underside surface. Also shown in the figure are the locations of pore-pressure transducers placed within the soil: the x–z coordinates of the sensor locations are presented in table 2. The water-pressure transducer is installed adjacent to the model, level with the soil surface and parallel to the direction of flow: the location is indicated with filled dots in figure 2*d*. Total normal-stress transducers are placed with the pore-pressure sensors at X3Z1 and X3Z4. From hereinafter, we identify the sensor locations by the coordinates shown in figure 4: namely, X for the longitudinal locations and Z for the vertical locations. Moreover, for brevity, we call the case with no impermeable layer the *Flat Beach* experiment and call the case with the impermeable layer installed the *Layer* experiment.

**Table 2.** Pore-pressure sensor locations (x:z in mm) based on the coordinate system shown in figure 4.

	X1	X2	X3	X4
Z1	142:−48	224:−45	374:−49	439:−50
Z2	142:−94	226:−92	373:−94	445:−94
Z3	146:−143	232:−144	375:−142	
Z4	148:−214		375:−214	

## 4. Results

All data shown in this section are presented at the model scale. The field scale values are found by multiplying the model values for lengths and time by N (=40), and by using a one-to-one ratio for velocity and pressure. For the runup stage, based on the sensor response and the leading surge line observed with the high-speed video, the flow is sufficiently uniform across the surface of the soil specimen. The drawdown is also uniform laterally across the soil specimen.

**Figure 5.**
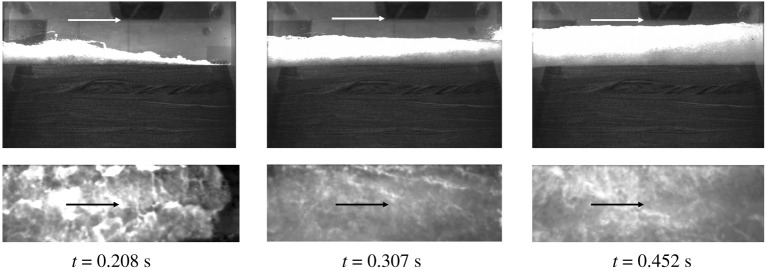
Snapshots of the video footage for the initial runup process. The video camera was installed above the soil box looking downward (figure 2*d*,*e*). The upper part of the images is the side view through the mirror and observation window; the lower part is the top view. The arrow indicates the direction of flow.

Figure 5 shows snapshots of the video footage for the Layer experiment. The bright portion in the image is the water flow dyed with fluorescein. The view at t=0.208 s captures the moment just prior to the leading surge tongue reaching the downstream end of the specimen box. At t=0.307 s, we see a small splash from the initial impact with the end wall of the container. It is seen that sediments are picked up and entrained in the flow, as anticipated by the sufficiently large value of Shields number Θ≈0.9 (table 1): as we discussed in §3, the critical value of Θ that causes incipient sediment motion is less than $O(10^{-1})$. Nonetheless, change in soil-surface profile was found to be negligible, less than 3 mm in the area over the impermeable layer according to Exton [30]. The flow continues (t=0.452 s) without a sign of backup flow: the continual discharge is filling the space around the specimen box (figure 2*d*).

**Figure 6.**
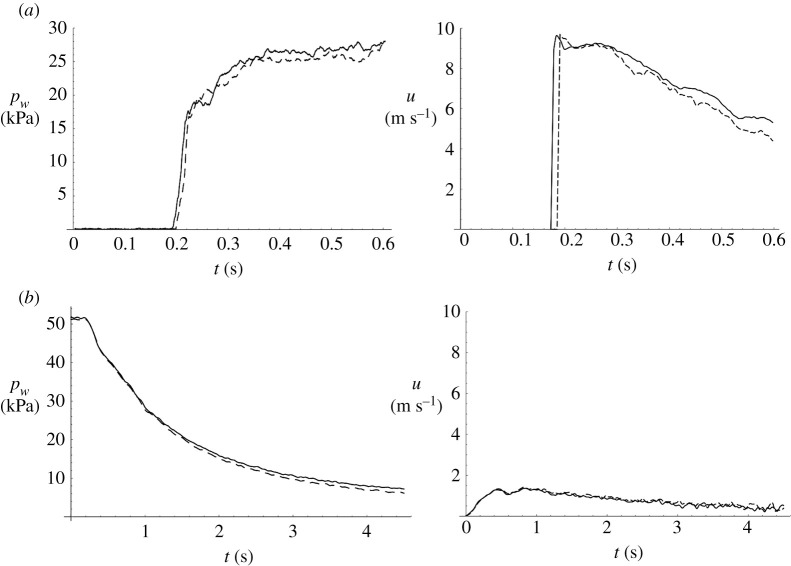
Temporal variations of water pressure $p_{w}$ on the soil surface and flow velocity u for two experiments: solid line, the Layer case (with impermeable layer); dashed line, the Flat Beach case (without impermeable layer). (*a*) Runup process; (*b*) drawdown process.

Because the flow conditions are carefully controlled by the geometry of the apparatus, the volume of water used and the centripetal acceleration, the experimental flow conditions are repeatable. The conditions of the two experiments (Layer and Flat Beach) are nearly identical, as demonstrated in the time series of water pressure during the runup and drawdown stages in figure 6. There is a small, observable difference in the arrival time of the surge front as well as in the variations of the water surface. Towards the end of the drawdown stage there is also a small difference in water height. The water velocity between the experiments is also nearly identical, as shown in figure 6. Note that the runup flow speeds are measured using high speed video (Photron AX-100) and estimated using Farnebäck’s [35] optical-flow algorithm applied to the top view of the flow, as shown in the bottom panels of figure 5. Figure 6*a* shows that during runup, the maximum flow velocity is approximately $10\;{\text{m s}}^{-1}$ and occurs at the leading edge of the surge front; the velocity gradually reduces to around 5–6 m s${\;}^{-1}$ at t=0.6 s. During drawdown (figure 6*b*), the average flow speed is between 1 and $2\;{\text{m s}}^{-1}$. (Note that, because the Farnebäck algorithm tracks aeration in the flow, it cannot be applied to the video to determine the drawdown velocity. Instead, we estimated the flow speed based on the conservation of volume using the measured data of receding water depth.) The measured velocities confirm that the target flow speeds represent realistic tsunami inundation processes of the field (table 1).

For runup, we first focus on how the soil responds to the initial surging process as the leading tongue runs across the soil specimen box: in the duration of 0<t<0.6 s (note that the runup gate opens at time t=0). After t=0.6 s, the incoming flow is affected by backflow resulting from the enclosed container. Later analysis will include the backflow as it induces secondary soil response. No such disturbance occurred in the drawdown stage, so we will analyse for the entirety of the drawdown stage, from 0<t<4.5 s (the time origin of the drawdown is set at the instance when the drawdown gate opens). Recall that the duration of 0<t<0.6 s in the model runup is equivalent to the duration of 24 s in the field, and the model drawdown for 0<t<4.5 s is equivalent to the duration of 180 s in the field.

### (a) Runup

**Figure 7.**
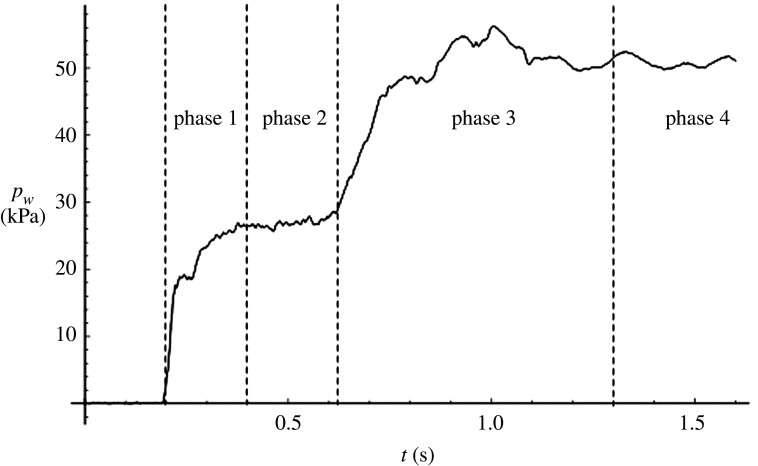
Temporal variation of water pressure $p_{w}$ at X3 in the runup process for the Layer case. The water pressure was measured with the pressure transducer placed on the soil surface along the sidewall of the soil specimen box: see figure 2*d*. Phases 1 and 2 represent the increase in inundation depth due to the initial surge; phase 3 shows the increase in water depth by the backup flow resulted from the finite space of the runup water storage as seen in figure 2*d*; phase 4 represents the equilibrium state after the full inundation is reached.

The tsunami runup stage can be divided into four phases, as shown in figure 7. In phase 1, there is a sudden increase in overburden loading by the surging front at a rate of approximately 840 kPa/s (commencing at t∼0.2 s). In phase 2, there is near constant loading during the flooding process (commencing at t∼0.4 s). Phase 3 exhibits a gradual increase in loading due to backflow caused by the limited volume of the runup-water storage (figure 2*d*) at an approximate rate of $160\;{\text{kPa s}}^{-1}$ (commencing at t∼0.6 s). Finally, in phase 4, an equilibrium static state is established (roughly t≳1.3 s).

**Figure 8.**
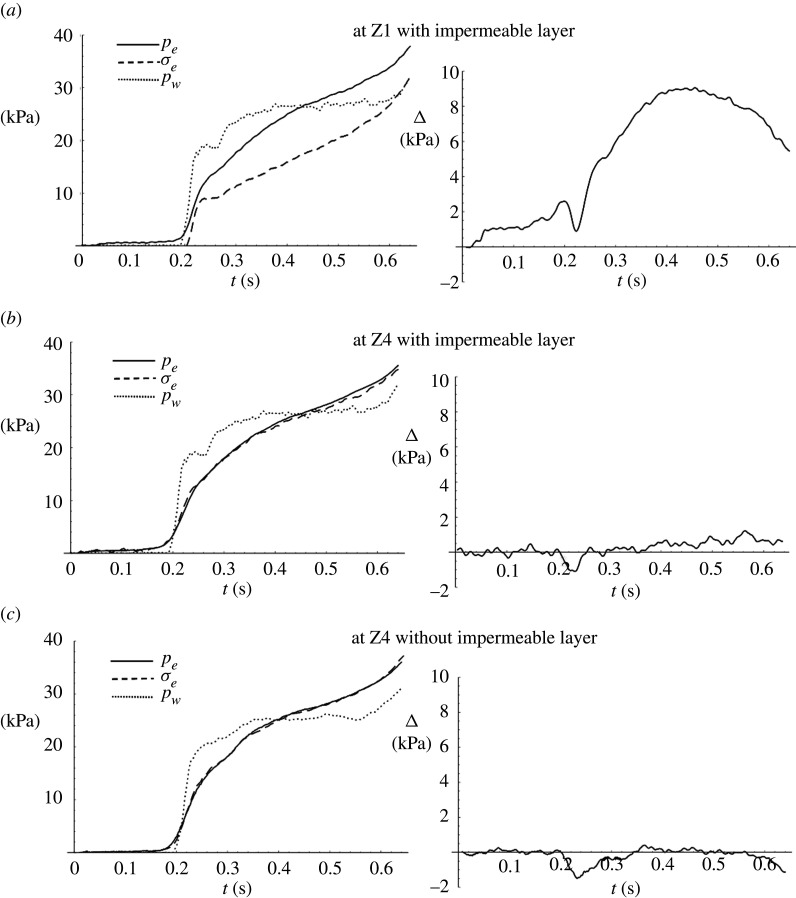
Soil response at X3 during the initial runup stage (phases 1 and 2 as denoted in figure 7.) The left panels show the temporal variations of excess pore pressure $p_{e}$ (solid line), excess total stress, $\sigma_{e}$ (dashed line) and water pressure on the soil surface $p_{w}$ (dotted line). The right panels show the difference between the excess pore pressure and the excess total stress, $\Delta =p_{e}-\sigma_{e}$, (*a*) at the underside of the impermeable layer (Z1) in the layer experiment; (*b*) at the bottom of the soil specimen (Z4) in the Layer experiment; (*c*) at the bottom of the soil specimen without impermeable layer (Z4) in the Flat Beach experiment. The critical values of Δ for vanishing effective vertical stress at Z1 and Z4 are 13.7 and 83.4 kPa, respectively.

Figure 8 shows the phase 1 response at X3 due to the sudden load increase at the arrival of the surge front. The left panels show the temporal variations of water pressure $p_{w}$ on the soil surface, the excess total stress $\sigma_{e}$ and the excess pore pressure $p_{e}$. Note that $\sigma_{e}$ is obtained from the data of the total normal-stress transducers, and $p_{e}$ is obtained from the data of the pore-pressure transducers. The right panels show the difference between excess pore pressure and excess total stress, $\Delta =p_{e}-\sigma_{e}$, at three locations: (a) at Z1, the underside of the impermeable layer in the Layer experiment; (b) at Z4, the bottom of the soil specimen, also in the Layer experiment; (c) at Z4, the bottom of the soil specimen without impermeable layer in the Flat Beach experiment. According to (2.4), Δ represents the degree of soil instability. The soil response is caused by the sudden water-surface increase in phase 1 ($∼840\;{\text{kPa s}}^{-1}$). This loading is extremely transient and therefore, interpreting the soil response based on our knowledge of the equilibrium state would be misleading.

It is clear from figure 8*b*,*c* that the excess pore pressure and the excess total stress behave very similarly (Δ∼0) at the bottom of the soil specimen regardless of the presence of the impermeable layer. On the other hand, figure 8*a*, right shows that there is a substantial increase in Δ, approximately 9.15 kPa, immediately beneath the impermeable layer. This is 67% of the critical value of $\Delta (=(\rho_{\text{sat}}-\rho )Gz=13.7\;\text{kPa}$) at Z1 that causes the effective stress to vanish: see equation (2.4). (Note that the critical Δ is computed considering the presence of the impermeable layer.) Figure 8*a*, left indicates that the increase in Δ is a result of a delay in the excess total stress response to the overburden load $p_{w}$. This behaviour seems to be confined to the region just beneath the impermeable layer because no influence is detected at the deeper location in the same soil specimen (figure 8*b*, right). A possible cause of this localized and transient behaviour is dislocation of the soil lattice caused by the impermeable layer due to the sudden impulse of substantial overburden load. This appears to resemble the mechanism of blast-induced liquefaction [36,37], although the impulse in the present case is made by a plane (impermeable layer), not a point source. Blast-induced liquefaction also takes place at a shorter duration than the present case.

**Figure 9.**
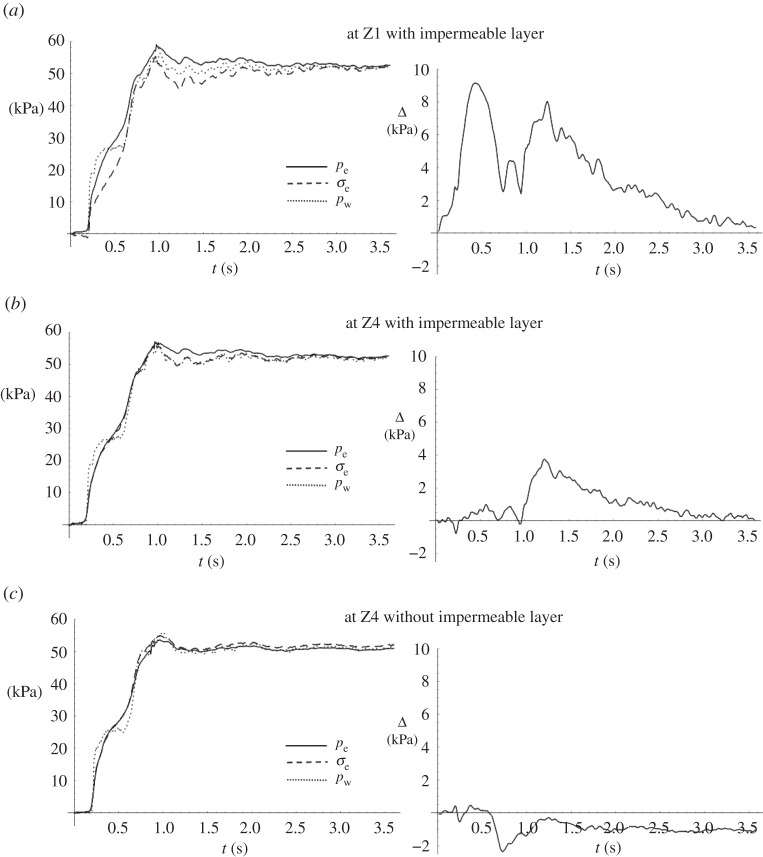
Soil response at X3 during the entire runup process. The left panels show the temporal variations of excess pore pressure $p_{e}$ (solid line), excess total stress $\sigma_{e}$ (dashed line) and water pressure on the soil surface $p_{w}$ (dotted line). The right panels show the difference between the excess pore pressure and the excess total stress, $\Delta =p_{e}-\sigma_{e}$, (*a*) at the underside of the impermeable layer (Z1); (*b*) at the bottom of the soil specimen (Z4); (*c*) at the bottom of the soil specimen without impermeable layer. The critical values of Δ for vanishing effective stress at Z1 and Z4 are 13.7 kPa and 83.4 kPa, respectively.

We now include the backup-flow phase in the soil response analysis. Figure 9 shows the soil response at X3 as was shown in figure 8 but for a longer duration; plotting the longer duration reveals a secondary increase in Δ that occurs in phase 3 (figure 7). In phase 3, the water pressure on the soil surface increases gradually in comparison with phase 1. Figure 9*c* shows that when there is no impermeable layer in the soil, the pressure on the soil surface $p_{w}$, the excess total stress $\sigma_{e}$ and the excess pore pressure $p_{e}$ essentially coincide as expected for a quasi-equilibrium process. By contrast, when the impermeable layer is present, the pore pressure exceeds the excess total stress; see the right panels of figures 9*a*,*b*. The difference Δ reaches a maximum at the end of phase 3 (t=1.2∼1.3 s), then gradually converges at t∼3.5 s at both depths (Z1 and Z4), indicating that the equilibrium state is reached within the soil. Note that equilibrium within the soil is reached after the surface flow reaches equilibrium.

At the underside of the impermeable layer, figure 9*a*, left shows that the excess pore pressure $p_{e}$ exceeds both the excess total stress $\sigma_{e}$ and water pressure load $p_{w}$ on the soil surface after the cessation of the backup flooding at t∼1 s. As shown in figure 9*a*, right, the excess total stress response is lower than the increase in pore pressure at the underside of the layer, i.e. Δ>0. The increase in overburden stress in phase 3, although less rapid than that in phase 1, must be sufficiently fast to cause another process of soil lattice dislocation, which contributes to the creation of a substantial value of Δ: the maximum value is Δ=8.12 kPa, at t=1.24 s (recall that the critical value of Δ indicating zero effective stress and thus soil liquefaction is 13.7 kPa at Z1). In this case, however, the effect of soil lattice dislocation/rearrangement is prolonged for a duration longer than the phase-1 response presented in figure 8, and gradually approaches its equilibrium state: it takes more than 2 s. Furthermore, this effect propagates to the bottom of the soil specimen, as shown in figure 9*b*, right. These behaviours are different from those that occurred at the initial impact of the surge front (t≲0.6 s) presented in figure 8.

At the bottom of the soil specimen with the impermeable layer, figure 9*b*, left shows that the excess total stress $\sigma_{e}$ coincides with the overburden stress (pressure on the soil surface, $p_{w}$) for t>1 s. This represents a quasi-equilibrium state for a stable soil lattice at Z4. However, the excess pore pressure $p_{e}$ exceeds the excess total stress $\sigma_{e}$: this is caused by the impedance of the impermeable layer, which delays pore-pressure dissipation. The excess pore pressure exceeds both the overburden stress and the excess total stress during the entire process in phases 3 and 4. This behaviour is similar to the phenomenon called ‘overpressure,’ which is the state of pore pressure exceeding the hydrostatic condition and is commonly discussed in the field of geology [38–41]. In the field of geology, abnormally high pore pressure can develop where burial of sediments is swift and the permeability is low, such that pore fluids cannot escape rapidly enough; this condition causes the pore pressure to increase as overburden increases. This process is called ‘disequilibrium compaction’, a phenomenon known to occur at a depth of 1∼2 km. The overpressure caused by disequilibrium compaction dissipates slowly (over many years) due to slow fluid movement. The present case is like the overpressure phenomenon, but the time scale is much shorter (seconds to minutes in the field time scale) and the spatial scale is much smaller (metres in depth in the field scale). Rapidly transient overburden loading creates a condition similar to the ‘overpressure’ under the impermeable layer. The disequilibrium state must be corrected by the pore-pressure dissipation around the outer edge of the impermeable layer. Our laboratory data shown in figure 9 support the conjecture of the occurrence of the overpressure-like phenomenon under the overburden load of tsunami-like inundation.

### (b) Drawdown

**Figure 10.**
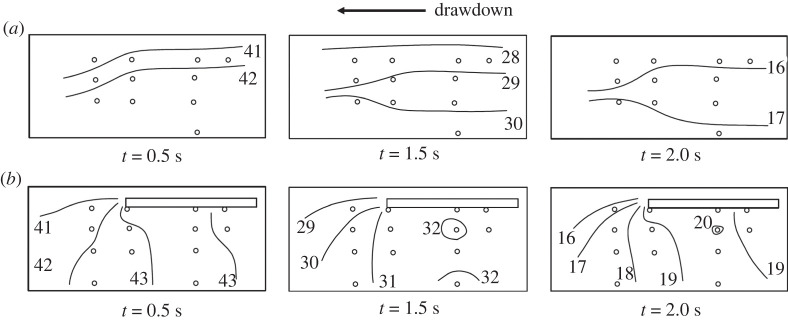
Isobaric contour plots of excess pore pressure for three instances during the drawdown stage of (*a*) the Flat Beach experiment and (*b*) the Layer experiment. The excess pore-pressure field in (*b*) is notably altered from the Flat Beach case in (*a*), with a locally high-pressure region forming beneath the layer. The locations of pore-pressure sensors are shown by open dots: the coordinates are listed in table 2. Note that the data points are sparse, therefore the isobaric contours are estimates and limited in resolution.

For the drawdown process, we first present the overall pore-pressure field at the centreline of the model in the form of isobaric contour plots in figure 10 for three instances after opening the drawdown gate: t=0.5,1.0 and 2.0 s. The plots are based on interpolations of the pore-pressure data using a mesh created with Delaunay triangulation [42]. The data are not extrapolated to the container boundaries; however, we consider the fact that the boundaries, except the soil surface, are impermeable and hence have a no-flux condition. The data points are sparse, and therefore the isobaric contours are limited in resolution. As seen in figure 10 for the Flat Beach case, the pressure release within the soil is fairly uniform across the soil specimen. On the other hand, the pressure release for the Layer case is notably altered by the presence of the impermeable layer. Relatively uniform pressures are retained underneath the layer, and a locally high region of pore pressure emerges under the layer.

**Figure 11.**
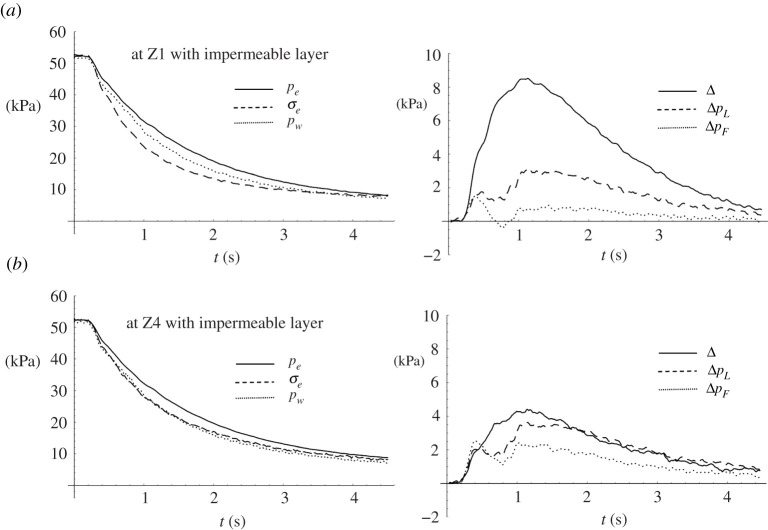
Soil response during the drawdown process at X3. The left panels show the temporal variations of excess pore pressure $p_{e}$ (solid line), excess total stress $\sigma_{e}$ (dashed line) and water pressure on the soil surface $p_{w}$ (dotted line). The right panels show the difference between the excess pore pressure and the excess total stress, $\Delta =p_{e}-\sigma_{e}$ (solid line); the difference between the excess pore pressure and the water pressure on the soil surface for the Layer case, $\Delta p_{L}=p_{e}-p_{w}$ (dashed line); the difference $\Delta p_{F}=p_{e}-p_{w}$ for the situation for the Flat Beach case (dotted line). (*a*) at the underside of the impermeable layer Z1 and (*b*) at the bottom of the soil specimen Z4. The critical values of Δ for vanishing effective vertical stress at Z1 and Z4 are 13.7 and 83.4 kPa, respectively.

The effects of the impermeable layer can be further explored and shown in figure 11, of which the left panels show the temporal variations of water pressure on the soil surface $p_{w}$, the excess total stress $\sigma_{e}$ and the excess pore pressure $p_{e}$ at X3 for the Layer experiment at two depths: Z1 and Z4. The right panels show the differences between the excess pore pressure and the excess total stress for the Layer case, $\Delta =p_{e}-\sigma_{e}$; the difference between the excess pore pressure and the water pressure on the soil surface for the Layer case, $\Delta p_{L}=p_{e}-p_{w}$; and the difference for the Flat Beach case, $\Delta p_{F}=p_{e}-p_{w}$. At the bottom of the soil specimen (Z4) for the Layer case, figure 11*b*, left shows that the change in water pressure on the soil surface closely coincides with the excess total stress. This is anticipated as for a quasi-equilibrium state of a stable soil lattice structure. That is not the case, however, at the underside of the impermeable layer (Z1), as shown in figure 11*a*, left. The excess total stress reduces at a faster rate than the reduction in water pressure on the soil surface. This must be caused by soil-grain dislocation due to the swift reduction of the overburden loading.

During drawdown, the downward gradients of pore pressure, which generate upward pressure forces, are developed: note that this phenomenon has been reported previously by Yeh *et al.* [4]. The quantitative process of this behaviour is exhibited by the dashed line ($\Delta p_{L}$) for the Layer case and by the dotted line ($\Delta p_{F}$) for the Flat Beach case in figure 11, right at both Z1 and Z4. The presence of the impermeable layer creates the condition of higher pore pressures than that for the case with no layer. This is because the presence of the layer is a barrier to drainage and therefore causes a delay in the dissipation of pore pressure. While figure 11, right shows that the difference in excess pore pressure and surface water pressure (for both $\Delta p_{L}$and $\Delta p_{F}$) at the deep location (Z4) is greater than that at the shallow location (Z1), the excess pore-pressure gradient is greater at the shallow location: hence less stable according to (2.5).

The maximum value of $\Delta (=p_{e}-\sigma_{e})$ in figure 11*a*, right is 8.51 kPa at t=1.13 s, which is approximately 60% of the critical value (=13.7 kPa at Z1) needed for the effective stress to vanish. The rapid drawdown associated with tsunamis can induce potentially substantial soil instability under the impermeable layer. As discussed in §2, the soil tends to become unstable when the effective stress becomes approximately one-half of the equilibrium state [6,28]. While disturbance of the soil is not observed at the surface in the experiment, it is likely that the soil supporting the impermeable layer from the underneath is substantially weakened.

## 5. Conclusion

Many observations of post-tsunami surveys indicate that soil instability plays a substantial role in causing damage to coastal infrastructure. Yet, soil response to tsunami loading is not adequately understood. We present an experimental attempt to explore fundamental mechanics of soil instability induced by tsunami-like loading. To achieve a controlled laboratory environment, we utilize a centrifuge apparatus that enables us to enhance the body force and viscous force to reduce scale effects. The apparatus realistically creates tsunami runup and drawdown processes in a single experiment and allows us to obtain quantitative measurements of the fluid flow velocities, depths and pressures, as well as measurements of soil response in terms of pore pressures and total soil stresses. The present hypergravity experiments represent a field-scale runup flow speed of approximately $10\;{\text{m s}}^{-1}$, inundation depth of 5 m and drawdown period of approximately 2 min with a flow speed of 1–$2\;{\text{m s}}^{-1}$. Such flow conditions are representative of a typical tsunami event. We study two cases: a homogeneous soil specimen with a flat, horizontal surface, and the same soil condition with an impermeable rigid horizontal layer embedded near the soil surface. The latter case is considered important based on the field observations: see equation figure 1, for example. The results of the present experiments yield unique measurements of soil response to transient tsunami loading.

In the present experiments, soil response is monitored by the variations of excess pore pressures and excess total stresses. As an indicator of soil instability, we calculate the difference between the excess pore pressure and the excess total stress, $\Delta =p_{e}-\sigma_{e}$. According to (2.4), the soil loses intergrain resistance when $\Delta \to (\rho_{\text{sat}}-\rho )Gz$. Furthermore, as we stated in §2, Sumer *et al.* [28] and Tonkin *et al.* [6] suggest that soil instability may occur earlier than the criterion under dynamic loading conditions. While soil liquefaction was not observed visually in the present experiments, the data evidently indicate that the rapid runup and drawdown stages cause substantial soil response towards instability during both the runup phase and the drawdown phase.

In the runup phase, it is thought that increase in water level after passing the surge front results in a positive pressure gradient in the soil, hence enhanced soil stability. However, our laboratory results show otherwise when the impermeable layer is present. Immediately after the tsunami surge arrives, the sudden pressure impact on the soil surface causes dislocation of soil grains at the underside of the impermeable layer (Figure 8*a*). The excess total stress temporarily becomes lower than the excess pore pressure, leading to instability: our results show a 67% reduction of the effective body force. The soil stability quickly recovers afterward: the recovery commences around 0.5 s (figure 8*a*, right), which is about 20 s in field time scale. It is noted that this ‘impulse-induced instability’ is confined in the region near the underside of the impermeable layer; the soils in the deeper location are not affected.

During the later runup stage, an additional increase in overburden load occurs due to the backup flow (figure 9). This secondary load is slower than the initial surging load, and also induces a difference in the excess pore pressure and the excess total stress, Δ, resulting in a reduction of the effective body force. Unlike the localized and short-lived instability associated with the rapid initial loading, the reduction in effective stress under the impermeable layer affects the deeper part of the soil specimen, and is prolonged for a longer duration until gradually approaching its equilibrium state. At the underside of the impermeable layer, this is a result of the lower excess total stress and the higher excess pore pressures than those in the quasi-equilibrium state. At the bottom of the soil specimen, the reduction of the effective stress is caused by the enhanced and prolonged pore pressure. It appears that this behaviour resembles the mechanism of so-called ‘overpressure‘ or ‘disequilibrium compaction’, which is discussed in the field of geology at a much longer time and larger depth scale.

During drawdown, the downward gradients of pore pressure, which generate upward pressure forces, are developed due to the rapid relief of the overburden load. While there have been a few laboratory studies to address this mechanism of soil instability [4,5], reported herein are the first quantitative measurements of pore pressures and vertical soil stresses under realistic tsunami loading scales. This is a notable advantage of centrifuge experiments in terms of stress states. The soil instability effect is substantially enhanced with the presence of the impermeable layer: our results show a 60% reduction of the effective body force (figure 11). The data illustrate that rapid drawdown associated with tsunamis can induce substantial soil instability under the impermeable layer. While instability of the soil may not be observed at the surface, soil supporting structures and pavements may be weakened substantially.

Soil instability mechanisms revealed in the present study advance understandings of damaging effects caused by rapid tsunami-like inundation processes. The findings should contribute to yield improvements in design, construction and retrofit of infrastructure and buildings in potentially vulnerable flood zones by extreme events.

## Data accessibility

All data used in this publication are openly available at https://oregonstate.box.com/v/RSPA-Exton-Yeh-2021. Also note that this research was funded under the NSF-supported Natural Hazards Engineering Research Infrastructure (NHERI). All data produced under NHERI awards are published on the public repository, DesignSafe-CI: https://www.designsafe-ci.org
